# A Verified Implementation of the Berlekamp–Zassenhaus Factorization Algorithm

**DOI:** 10.1007/s10817-019-09526-y

**Published:** 2019-06-17

**Authors:** Jose Divasón, Sebastiaan J. C. Joosten, René Thiemann, Akihisa Yamada

**Affiliations:** 1 University of La Rioja, Logroño, Spain; 2 University of Innsbruck, Innsbruck, Austria

**Keywords:** Factor bounds, Hensel lifting, Isabelle/HOL, Local type definitions, Polynomial factorization, Theorem proving

## Abstract

We formally verify the Berlekamp–Zassenhaus algorithm for factoring square-free integer polynomials in Isabelle/HOL. We further adapt an existing formalization of Yun’s square-free factorization algorithm to integer polynomials, and thus provide an efficient and certified factorization algorithm for arbitrary univariate polynomials. The algorithm first performs factorization in the prime field $\mathrm {GF}(p){}$ and then performs computations in the ring of integers modulo $p^k$, where both *p* and *k* are determined at runtime. Since a natural modeling of these structures via dependent types is not possible in Isabelle/HOL, we formalize the whole algorithm using locales and local type definitions. Through experiments we verify that our algorithm factors polynomials of degree up to 500 within seconds.

## Introduction

Modern algorithms to factor univariate integer polynomials—following Berlekamp and Zassenhaus—first preprocesses the input polynomial to extract the content and detect duplicate factors. Afterwards, the main task is to factor primitive square-free integer polynomials, first over prime fields $\mathrm {GF}(p)$, then over quotient rings ${\mathbb {Z}}/{p^k}{\mathbb {Z}}$, and finally over integers ${\mathbb {Z}}$ [5, 8]. Algorithm 1 illustrates the basic structure of such a method for factoring polynomials.^1^
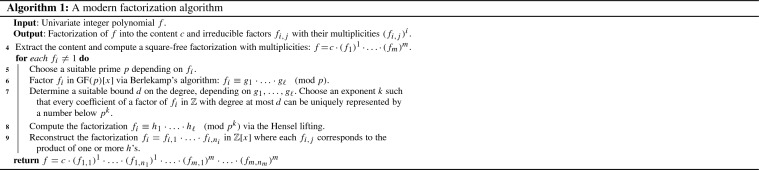


In earlier work on algebraic numbers [31] we implemented Algorithm 1 in Isabelle/HOL [29]. There, however, the algorithm was not formally proven correct and thus followed by certification, i.e., a validity check on the result factorization. Moreover, there was no guarantee on the irreducibility of resulting factors. During our formalization we indeed found an error in the implementation of Line 7 of this earlier work. Since in several experiments with algebraic numbers this error was not exposed, this clearly shows the advantage of verification over certification.

In this work we fully formalize the correctness of our implementation. It delivers a factorization into the content and a list of irreducible factors.

### Theorem 1

(Factorization of Univariate Integer Polynomials) 
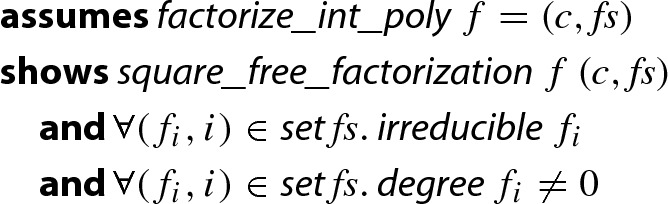


Here,  means that $f = c \cdot f_1^{m_1+1} \cdot \ldots \cdot f_n^{m_n+1}$, *c* is a constant, each $f_i$ is square-free, and $f_i$ and $f_j$ are coprime whenever $i \ne j$.

To obtain Theorem 1 we perform the following tasks.

- In Sect. 3 we introduce three Isabelle/HOL definitions of ${\mathbb {Z}}/{m}{\mathbb {Z}}$ and $\mathrm {GF}(p)$. We first define a *type* to represent these domains, which allows us to reuse many algorithms for rings and fields from the Isabelle distribution and the AFP (Archive of Formal Proofs). At some points in our development, however, the type-based setting becomes too restrictive. Hence we also introduce the second *integer representation*, which explicitly applies the remainder operation modulo *m*. For efficient implementation we also introduce the third representation, which allows us to employ machine integers [24] for reasonably small *m*. Between the representations we transform statements using *transfer* [15] and *local type definitions* [21].
- The first part of the algorithm is square-free factorization over integer polynomials. In Sect. 4 we adapt Yun’s square-free factorization algorithm [32, 35] from ${\mathbb {Q}}$ to ${\mathbb {Z}}$.
- The prime *p* in step 5 must be chosen so that $f_i$ remains square-free in $\mathrm {GF}(p)$. Therefore, in Sect. 5 we prove the crucial property that such a prime always exists.
- In Sect. 6, we formalize Berlekamp’s algorithm, which factors polynomials over prime fields, using the type-based representation. Since Isabelle’s code generation does not work for the type-based representation of prime fields, we follow the steps presented in Sect. 3 to define a record-based implementation of Berlekamp’s algorithm and prove its soundness.
- In Sect. 7 we formalize Mignotte’s factor bound and Graeffe’s transformation used in step 7, where we need to find bounds on the coefficients and degrees of the factors of a polynomial. During this formalization task we detected a bug in our previous oracle implementation, which computed improper bounds on the degrees of factors.
- In Sect. 8 we formalize Hensel’s algorithm, lifting a factorization modulo *p* into a factorization modulo $p^k$. The basic operation there is lifting from $p^i$ to $p^{i+1}$, which we formalize in the type-based setting. Unfortunately, iteratively applying this basic operation to lift *p* to $p^k$ cannot be done in the type-based setting. Therefore, we remodel the Hensel lifting using the integer representation. We moreover formalize the *quadratic* Hensel lifting and consider several approaches to efficiently lift *p* to $p^k$.
- Details on step 9 are provided in Sect. 9 where we closely follow the brute-force algorithm as it is described by Knuth [18, p. 452]. Here, we use the same representation of polynomials over ${\mathbb {Z}}/{p^k}{\mathbb {Z}}$ as for the Hensel lifting.
- In Sect. 10 we illustrate how to assemble all the previous results in order to obtain the verified  algorithm. This includes some optimizations for improving the runtime of the algorithm, such as the use of reciprocal polynomials and Karatsuba’s multiplication algorithm.
- Finally, we compare the efficiency of our factorization algorithm with the one in Mathematica 11.2 [34] in Sect. 11 and give a summary in Sect. 12.

Since the soundness of each sub-algorithm has been formalized separately, our formalization is easily reusable for other related verification tasks. For instance, the polynomial-time factorization algorithm of Lenstra et al. [23] has been verified [11], and that formalization could directly use the results about steps 4–8 of Algorithm 1 from this paper without requiring any adaptations.

Our formalization is available in the AFP. The following website links theorems in this paper to the Isabelle sources. Moreover, it provides details on the experiments.

https://doi.org/10.5281/zenodo.2525350

The formalization as described in this paper corresponds to the AFP 2019 version which compiles with the Isabelle 2019 release.

### Related Work

To our knowledge, the current work provides the first formalization of a modern factorization algorithm based on Berlekamp’s algorithm. Indeed, it is reported that there is no formalization of an efficient factorization algorithm over $\mathrm {GF}(p)$ available in Coq [4, Sect. 6, note 3 on formalization].

Kobayashi et al. [19] provide an Isabelle formalization of Hensel’s lemma. They define the valuations of polynomials via Cauchy sequences, and use this setup to prove the lemma. Consequently, their result requires a ‘valuation ring’ as a precondition in their formalization. While this extra precondition is theoretically met in our setting, we did not attempt to reuse their results, because the type of polynomials in their formalization (from HOL-Algebra) differs from the polynomials in our development (from HOL/Library). Instead, we formalize a direct proof for Hensel’s lemma. The two formalizations are incomparable: On the one hand, Kobayashi et al. did not restrict to integer polynomials as we do. On the other hand, we additionally formalize the quadratic Hensel lifting [36], extend the lifting from binary to *n*-ary factorizations, and prove a uniqueness result, which is required for proving Theorem 1. A Coq formalization of Hensel’s lemma is also available. It is used for certifying integral roots and ‘hardest-to-round computation’ [26].

If one is interested in certifying a factorization, rather than in a certified algorithm that performs it, it suffices to test that all the found factors are irreducible. Kirkels [17] formalized a sufficient criterion for this test in Coq: when a polynomial is irreducible modulo some prime, it is also irreducible in ${\mathbb {Z}}$. These formalizations are in Coq, and we did not attempt to reuse them, in particular since there are infinitely many irreducible polynomials which are reducible modulo every prime.

This work is a revised and extended version of our previous conference paper [10]. The formalization has been improved by adding over 7000 lines of new material, which are detailed through different sections of this paper. This new material has been developed to improve the performance of the verified factorization algorithm and includes among others:

- Integration of unsigned-32/64-bit integer implementation, cf. Sect. 3.
- Formalization of distinct-degree factorization and integration of it as an optional preprocessing step for Berlekamp’s factorization, cf. Sect. 6.3.
- Integration of Graeffe’s transformation for tighter factor bounds, cf. Sect. 7.
- Formalization of a fast logarithm algorithm, required for Graeffe’s transformation, cf. Sect. 7.
- Formalization of balanced multifactor Hensel lifting based on factor trees, cf. Sect. 8.
- Formalization of Karatsuba’s polynomial multiplication algorithm, cf. Sect. 10.
- Improvements on the GCD algorithm for integer polynomials, cf. Sect. 10.
- Integration of reciprocal polynomial before factoring, cf. Sect. 10.
- Overall, the runtime of our verified factorization algorithm has improved significantly. The new implementation is more than 4.5 times faster than the previous version [10] when factoring 400 random polynomials, and the new version is only 2.5 times slower than Mathematica’s factorization algorithm.

## Preliminaries

Our formalization is based on Isabelle/HOL. We state theorems, as well as certain definitions, following Isabelle’s syntax. For instance,  is the ring homomorphism from integers to type $\alpha$, which is of *class* . Isabelle’s type classes are similar to Haskell; a type class is defined by a collection of operators (over a single type variable $\alpha$) and premises over them. The type class  is provided by the HOL library, representing the algebraic structure of ring with a multiplicative unit. We also often use the extension of the above function  to polynomials, denoted by  . Isabelle’s keywords are written in  . Other symbols are either clear from their notation, or defined on their appearance. We only assume the HOL axioms and local type definitions, and ensure that Isabelle can build our theories. Consequently, a sceptical reader that trusts the soundness of Isabelle/HOL only needs to validate the definitions, as the proofs are checked by Isabelle.

We also expect basic familiarity with algebra, and use some of its standard notions without further explanation. The notion of polynomial in this paper always means univariate polynomial. Concerning notation, we write  for the leading coefficient of a polynomial *f* and $\mathsf {res}_{}(f,g)$ for the resultant of *f* and another polynomial *g*.

The derivative of a polynomial $f = \sum _{i=0}^n a_i x^i$ is $f' = \sum _{i=1}^n i a_i x^{i-1}$. A *factorization* of a polynomial *f* is a decomposition into irreducible *factors*$f_1,\ldots ,f_n$ such that $f = f_1 \cdot \ldots \cdot f_n$. The irreducibility of a ring element *x* is defined via divisibility (denoted by the binary relation  following Isabelle):1We also define the degree-based irreducibility of a polynomial *f* as2Note that (1) and (2) are not equivalent on integer polynomials; e.g., a factorization of $f = 10x^2-10$ in terms of (1) will be $f = 2 \cdot 5 \cdot (x-1) \cdot (x+1)$, where the prime factorization of the *content*, i.e., the GCD of the coefficients, has to be performed. In contrast, (2) does not demand a prime factorization, and a factorization may be $f = (10x-10)\cdot (x+1)$. Note that definitions (1) and (2) are equivalent on *primitive* polynomials, i.e., polynomials whose contents are 1, and in particular for field polynomials.

In a similar way to irreducibility w.r.t. (2), we also define that a polynomial *f* is *square-free* if there does not exist a polynomial *g* of non-zero degree such that $g^2$ divides *f*. In particular, the integer polynomial $2^2 x$ is square-free. A polynomial *f* is *separable* if *f* and its derivative $f'$ are coprime. Every separable polynomial is square-free, and in fields of characteristic zero, also the converse direction holds.

## Formalizing Prime Fields

Our development requires several algorithms that work in the quotient ring ${\mathbb {Z}}/{p^k}{\mathbb {Z}}$ and the prime field $\mathrm {GF}(p)$. Hence, we will need a formalization of these fundamental structures.

We will illustrate and motivate different representations of these structures with the help of a heuristic to ensure that two integer polynomials *f* and *g* are coprime [18, p. 453ff]: If *f* and *g* are already coprime in $\mathrm {GF}(p)[x]$ then *f* and *g* are coprime over the integers, too. In particular if *f* and its derivative $f'$ are coprime in $\mathrm {GF}(p)[x]$, i.e., *f* is separable modulo *p*, then *f* is separable and square-free over the integers. Hence, one can test whether *f* is separable modulo *p* for a few primes *p*, as a quick sufficient criterion to ensure square-freeness.

The informal proof of the heuristic is quite simple and we will discuss its formal proof in separate sections.

- If *f* is separable modulo *p*, then *f* is square-free modulo *p* (Sect. 3.1).
- If *f* is square-free modulo *p* then *f* is square-free in ${\mathbb {Z}}[x]$, provided that  and *p* are coprime (Sect. 3.2).
- Testing separability (i.e., coprimality) modulo *p* is implemented via the Euclidean algorithm in the ring $\mathrm {GF}(p)[x]$ (Sect. 3.3).

Moreover, we will describe the connection of the separate steps, which is nontrivial since these steps use different representations (Sect. 3.4).

### Type-Based Representation

The type system of Isabelle/HOL allows concise theorem statements and good support for proof automation [21]. In our example, we formalize the first part of the proof of the heuristic conveniently in a type-based setting for arbitrary fields, which are represented by a type variable $\tau$ with sort constraint  . All the required notions like separability, coprimality, derivatives and square-freeness are implicitly parametrized by the type.

#### Lemma 1


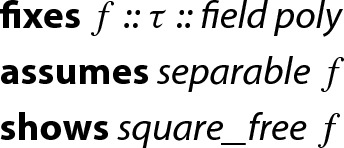


In order to apply Lemma 1 to a finite field $\mathrm {GF}(p)$ we need a type that represents $\mathrm {GF}(p)$. To this end, we first define a type to represent ${\mathbb {Z}}/{p}{\mathbb {Z}}$ for an arbitrary $p>0$, which forms the prime field $\mathrm {GF}(p)$ when *p* is a prime. Afterwards we can instantiate the lemma, as well as polymorphic functions that are available for  , e.g., the Gauss–Jordan elimination, GCD computation for polynomials, etc.

Since Isabelle does not support dependent types, we cannot directly use the term variable *p* in a type definition. To overcome the problem, we reuse the idea of the vector representation in HOL analysis [13]: types can encode natural numbers. We encode *p* as , i.e., the cardinality of the universe of a (finite) type represented by a *type* variable $\alpha$. The  keyword introduces a new type whose elements are isomorphic to a given set, along with the corresponding bijections. 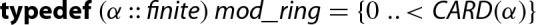 Given a finite type $\alpha$ with *p* elements,  is a type with elements 0, ..., $p - 1$. With the help of the lifting and transfer package, we naturally define arithmetic in  based on integer arithmetic modulo ; for instance, multiplication is defined as follows: 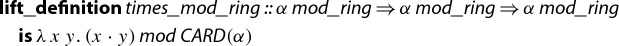 Here the  keyword applies the bijections from our type definition via  such that  is defined on  through a definition on the type of the elements of the set used in the  , namely natural numbers. It is straightforward to show that  forms a commutative ring: 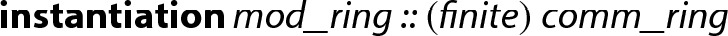 Note that  does not assume the existence of the multiplicative unit 1. If , then  is not an instance of the type class  , for which $0 \ne 1$ is required. Hence we introduce the following type class: 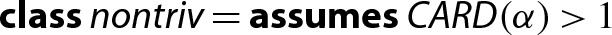 and derive the following instantiation:^2^
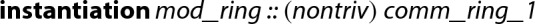


Now we enforce the modulus to be a prime number, using the same technique as above, namely introducing a corresponding type class. 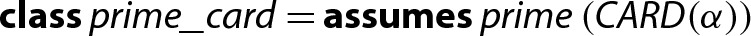


The key to being a field is the existence of the multiplicative inverse $x^{-1}$. This follows from Fermat’s little theorem: for any nonzero integer *x* and prime *p*,

$$\begin{aligned} x \cdot x^{p-2} \equiv x^{p-1} \equiv 1 \quad (mod p) \end{aligned}$$

that is,  if  is a prime. The theorem is already available in the Isabelle distribution for the integers, and we just apply the transfer tactic [15] to lift the result to . 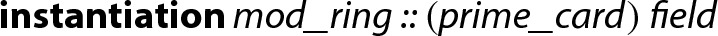 In the rest of the paper, we write  instead of .^3^

### Integer Representation

The type-based representation becomes inexpressive when, for instance, formalizing a function which searches for a prime modulus *p* such that a given integer polynomial *f* is separable modulo *p* and hence square-free modulo *p*. Isabelle does not allow us to state this in the type-based representation: there is no existential quantifier on types, so in particular the expression 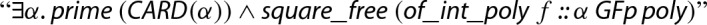 is not permitted.

Hence we introduce the second representation. This representation simply uses integers (type  ) for elements in ${\mathbb {Z}}/{m}{\mathbb {Z}}$ or $\mathrm {GF}(p)$, and uses  for polynomials over them. To conveniently develop formalization we utilize Isabelle’s *locale* mechanism [3], which allows us to locally declare variables and put assumptions on them in a hierarchical manner. We start with the following locale that fixes the modulus: 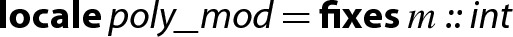 For prime fields we additionally assume the modulus to be a prime. 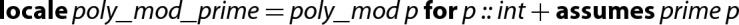 Degrees, divisibility and square-freeness for polynomials modulo *m* are defined by^4^
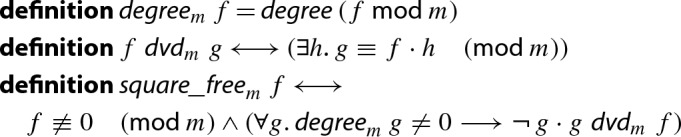


The integer representations have an advantage that they are more expressive than the typed-based ones. For instance, the soundness statement of the aforementioned function can be stated like “”. Another advantage of the integer representation is that one can easily state theorems which interpret polynomials in different domains like ${\mathbb {Z}}[x]$ and $\mathrm {GF}(p)[x]$. For instance, the second part of the soundness proof of the heuristic is stated as follows:

#### Lemma 2


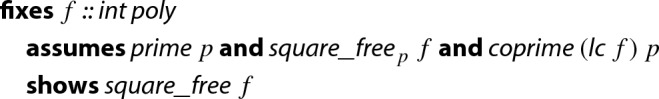


Note that there is no type conversion like  needed.

A drawback of this integer representation is that many interesting results for rings or fields are only available in the Isabelle library and AFP in type-based forms. To overcome the problem, we establish a connection between the type-based representation  and the locale  . This is achieved by first introducing the intermediate locale 
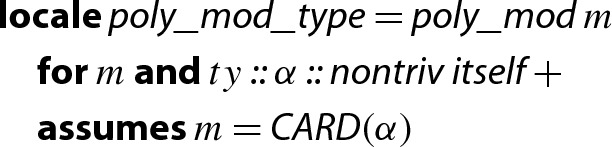
 for ${\mathbb {Z}}/{m}{\mathbb {Z}}$ and its sublocale for prime fields: 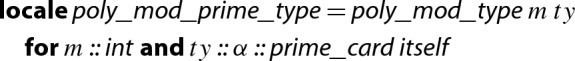 Second, we import type-based statements into these intermediate locales by means of *transfer* [15]. The mechanism allows us to translate facts proved in one representation into facts in another representation. To apply this machinery we first define the *representation* relation  describing when an integer polynomial represents a polynomial of type  . Then we prove a collection of *transfer rules*, stating the correspondences between basic notions in one representation and those in the other representation. For instance,

#### Lemma 3


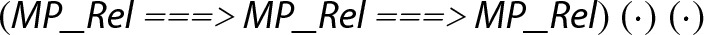


relates multiplication of polynomials of type  with multiplication of polynomials of type  . Concretely, it states that, if polynomials *f* and *g* of type  are related to polynomials ${{\overline{f}}}$ and ${{\overline{g}}}$ of type  respectively (via  ), then $f \cdot g$ is related to ${{\overline{f}}} \cdot {{\overline{g}}}$, again, via  . Note that the same syntax is used to represent the polynomial multiplication operation in both worlds (  and  ). The  symbol represents the relator for function spaces. That is, related functions map related inputs to related outputs. Then facts about rings and fields are available via transfer; e.g., from

#### Lemma 4


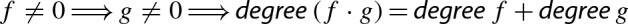


of standard library, we obtain

#### Lemma 5


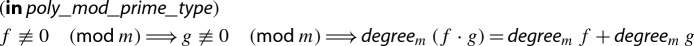


Finally, we migrate Lemma 5 from locale  to  . It is impossible to declare the former as a sublocale of the latter, since the locale assumption  can be satisfied only for certain $\alpha$. Instead, we see Lemma 5 from the global scope; then the statement is prefixed with assumption  . In order to discharge this assumption we use the *local type definition* mechanism [21], an extension of HOL that allows us to define types within proofs.

#### Lemma 6


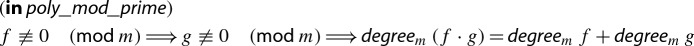


### Record-Based Implementation

The integer representation from the preceding section does not speak about how to implement modular arithmetic. For instance, although Lemma 3 can be interpreted as that one *can* implement multiplication of polynomials in ${\mathbb {Z}}/{m}{\mathbb {Z}} [x]$ by that over ${\mathbb {Z}}[x]$, there are cleverer implementations that occasionally take remainder modulo *m* to keep numbers small.

Hence, we introduce another representation.

#### Abstraction Layer

This third representation introduces an abstraction layer for the implementation of the basic arithmetic in ${\mathbb {Z}}/{m}{\mathbb {Z}}$ and $\mathrm {GF}(p)$, and builds upon it various algorithms over (polynomials over) ${\mathbb {Z}}/{m}{\mathbb {Z}}$ and $\mathrm {GF}(p)$. Such algorithms include the computation of GCDs, which is used for the heuristic when checking, for various primes *p*, whether the polynomial *f* is separable modulo *p*, i.e., the GCD of *f* and $f'$ in $\mathrm {GF}(p)[x]$ is 1 or not.

The following datatype, which we call *dictionary*, encapsulates basic arithmetic operations. Here the type variable $\rho$ represents Isabelle/HOL’s types for executable integers:  ,  , and  .^5^
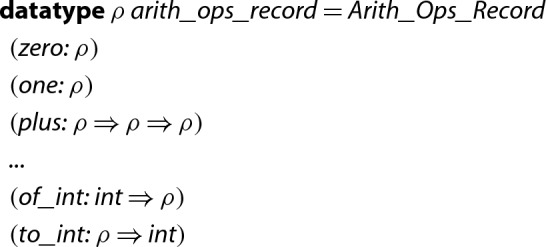


Given a dictionary *ops*, we build more complicated algorithms. For instance, following is the Euclidean algorithm for GCD computation, which is adjusted from the type-based version from the standard library.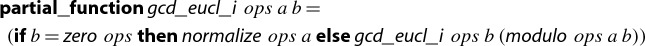 Here and often we use  [20], since  and others terminate only if *ops* contains a correct implementation of the basic arithmetic functions. Obviously, these algorithms are sound only if *ops* is correct. Correct means that the functions zero, plus etc. implement the ring operations and indeed form a euclidean semiring, a ring, or a field, depending on the algorithm in which the operations are used.

So we now consider proving the correctness of derived algorithms, assuming the correctness of *ops* in form of locales. The following locale assumes that *ops* is a correct implementation of a commutative ring $\tau$ using a representation type $\rho$, where correctness assumptions are formulated in the style of transfer rules, and locale parameter *R* is the representation relation. 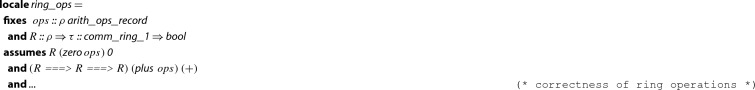


The second assumption just states that the output of the addition operation of the *ops* record ( *ops*) is related to the output of the addition operation $(+)$ of elements of type $\tau$ via *R*, provided that the input arguments are also related via *R*.

We need more locales for classes other than  . For instance, for the Isabelle/HOL class  , which admits the Euclidean algorithm, we need some more operations to be correctly implemented. 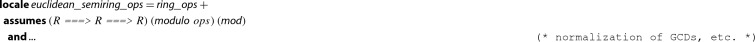 In this locale we prove the soundness of  , again in form of a transfer rule. The proof is simple since the definition of  is a direct translation of the definition of  .

##### Lemma 7


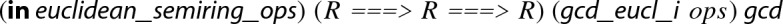


For class  moreover the inverse operation has to be implemented. Since in our application *p* is usually small, we compute $x^{-1}$ as $x^{p-2}$, using the binary exponentiation algorithm. 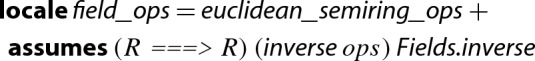


#### Defining Implementations

Here we present three record-based implementations of $\mathrm {GF}(p)$ using integers, 32-bit integers, and 64-bit integers. This means to instantiate $\tau$ by , and the representation type $\rho$ by  ,  , and  .

We first define the operations using  , which is essentially a direct translation of the definitions in Sect. 3.1. For example, $x \cdot y$ is implemented as  as in  , and the inverse of *x* is computed via $x^{p-2}$. The soundness of the implementation, stated as follows, is easily proven using the already established soundness proofs for the type-based version.

##### Lemma 8


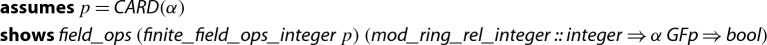


Hereafter,  denotes the dictionary of basic arithmetic operations for $\mathrm {GF}(p)$ (where the representation type $\rho$ should be clear), and  denotes the representation relation.

The implementations using  and  have the advantage that generated code will be more efficient as they can be mapped to machine integers [24]. It should be taken into account that they work only for sufficiently small primes, so that no overflows occur in multiplications: e.g., $65535 \cdot 65535 < 2^{32}$. The corresponding soundness statements look as follows, and are proven in a straightforward manner using the native words library [24].

##### Lemma 9


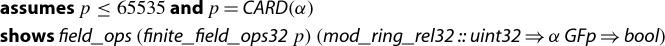


##### Lemma 10


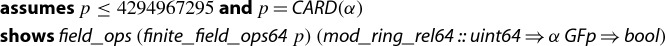


To obtain an implementation of GCD for polynomials over $\mathrm {GF}(p)$, we need further work: instantiating $\tau$ by . So we define a dictionary  implementing polynomial arithmetic. Here polynomials are represented by their coefficient lists: the representation relation between  and  is defined pointwise as follows. 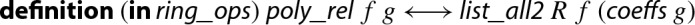 We define  by directly translating the implementations of polynomial arithmetic from the standard library; it is thus straightforward to prove the following correctness statement.

##### Lemma 11


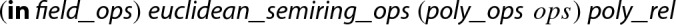


Finally we can instantiate Lemma 7 for polynomials as follows.

##### Lemma 12


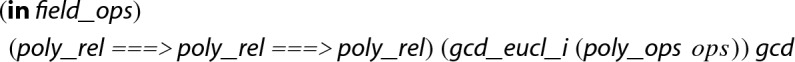


### Combination of Results

Let us shortly recall what we have achieved at this point. We formalized Lemma 1 in a type-based setting, and the type variable $\tau$ can be instantiated by the type , where the cardinality of $\alpha$ encodes the prime *p*. Moreover, we have a connection between square-freeness in $\mathrm {GF}(p)[x]$ and ${\mathbb {Z}}[x]$, all represented via integer polynomials in Lemma 2. Finally, we rewrote the type-based GCD-algorithm into a record-based implementation, and we provide three different records that implement basic arithmetic operations in $\mathrm {GF}(p)$ and $\mathrm {GF}(p)[x]$.

Let us now assemble all of the results. In the implementation layer we just define a test on separability of *f* using the existing functions like  from the implementation layers. In the following definition,  corresponds to the implementation of the one polynomial based on the  element provided by the arithmetic operations record. 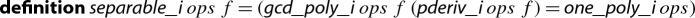 Since  requires as input the polynomial in the internal representation type $\rho$, we write a wrapper which converts an input *integer* polynomial into the internal type. Here,  heavily relies upon the function  from the arithmetic operations record. 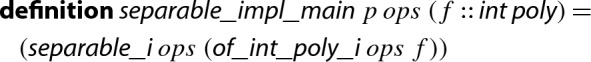


The soundness of this function as a criterion for square-freeness modulo *p* is proven in a locale which combines the locale  —*ops* is a sound implementation of —with the requirement that locale parameter *p* is equal to the cardinality of $\alpha$.

#### Lemma 13


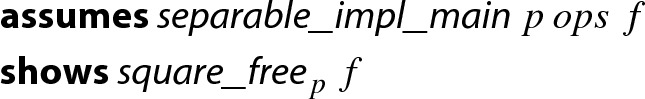


The proof goes as follows: Consider the polynomial . The soundness of  states that  and  are related by  . In combination with the soundness of  (via  ) we know that the GCD of *g* and $g'$ is 1, i.e., . Then Lemma 1 concludes  . Using the premise , we further prove , thus concluding .

Since we are still in a locale that assumes arithmetic operations, we next define a function of type  which is outside any locale. It dynamically chooses an implementation of $\mathrm {GF}(p)$ depending on the size of *p*.
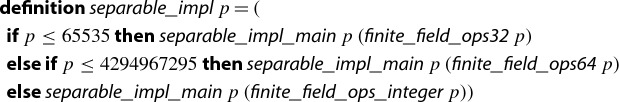


#### Lemma 14


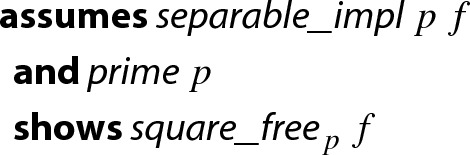


Although the soundness statement in Lemma 14 is quite similar to the one of Lemma 13, there is a major obstacle in formally proving it in Isabelle/HOL: Lemma 13 was proven in a locale which fixes a type $\alpha$ such that . In order to discharge this condition we have to prove that such a type $\alpha$ exists for every  . This claim is only provable using the extension of Isabelle that admits local type definitions [21].

Having proven Lemma 14, which solely speaks about integer polynomials, we can now combine it with Lemma 2 to have a sufficient criterion for integer polynomials to be square free.

The dynamic selection of the implementation of $\mathrm {GF}(p)$ in  —32-bit or 64-bit or arbitrary precision integers—is also integrated in several other algorithms that are presented in this paper. This improves the performance in comparison to a static implementation which always uses arbitrary precision integers, as it was done in our previous version [10], cf. Sect. 11.

## Square-Free Factorization of Integer Polynomials

Algorithm 1 takes an arbitrary univariate integer polynomial *f* as input. As the very first preprocessing step, we extract the content—a trivial task. We then detect and eliminate multiple factors using a square-free factorization algorithm, which is described in this section. As a consequence, the later steps of Algorithm 1 can assume that $f_i$ is primitive and square-free.

### Example 1

Consider the input polynomial $48 + 1128 x + 6579 x^2 - 1116 x^3 - 6042 x^4 + 5592 x^5 + 4191 x^6 - 2604 x^7 - 408 x^8 + 1080 x^9 + 300 x^{10}$. In step 4 of Algorithm 1 this polynomial will be decomposed into

$$\begin{aligned} 3 \cdot (\underbrace{4 + 47 x - 2 x^2 - 23 x^3 + 18 x^4 + 10 x^5}_f)^2. \end{aligned}$$

The square-free primitive polynomial *f* will be further processed by the remaining steps of Algorithm 1 and serves as a running example throughout this paper.

We base our verified square-free factorization algorithm on the formalization [32, Sect. 8] of Yun’s algorithm [35]. Although Yun’s algorithm works only for polynomials over fields of characteristic 0, it can be used to assemble a square-free factorization algorithm for integer polynomials with a bit of post-processing and the help of Gauss’ Lemma as follows: Interpret the integer polynomial *f* as a rational one, and invoke Yun’s algorithm. This will produce the square-free factorization $f = \ell \cdot f_{1,{\mathbb {Q}}}^1 \cdot \ldots \cdot f_{n,{\mathbb {Q}}}^n$ over ${\mathbb {Q}}$. Here, $\ell$ is the leading coefficient of *f*, and all $f_{i,{\mathbb {Q}}}$ are monic and square-free. Afterwards eliminate all fractions of each $f_{i,{\mathbb {Q}}}$ via a multiplication with a suitable constant $c_i$, i.e., define $f_{i,{\mathbb {Z}}} := c_i \cdot f_{i,{\mathbb {Q}}}$, such that $f_{i,{\mathbb {Z}}}$ is primitive. Define $c:= \ell \div (c_1^1 \cdot \ldots \cdot c_n^n)$. Then $f = c \cdot f_{1,{\mathbb {Z}}}^1 \ldots \cdot f_{n,{\mathbb {Z}}}^n$ is a square-free factorization of *f* over the integers, where *c* is precisely the content of *f* because of Gauss’ Lemma, i.e., in particular $c \in {\mathbb {Z}}$.

The disadvantage of the above approach to perform square-free factorization over the integers is that Yun’s algorithm over ${\mathbb {Q}}$ requires rational arithmetic, where after every arithmetic operation a GCD is computed to reduce fractions. We therefore implement a more efficient version of Yun’s algorithm that directly operates on integer polynomials. To be more precise, we adapt certain normalization operations of Yun’s algorithm from field polynomials to integer polynomials, and leave the remaining algorithm as it is. For instance, instead of dividing the input field polynomial by its *leading coefficient* to obtain a *monic* field polynomial, we now divide the input integer polynomial by its *content* to obtain a *primitive* integer polynomial. Similarly, instead of using the GCD for field polynomials, we use the GCD for integer polynomials, etc.

To obtain the soundness of the integer algorithm, we show that all polynomials $f_{\mathbb {Z}}$ and $f_{\mathbb {Q}}$ that are constructed during the execution of the two versions of Yun’s algorithm on the same input are related by a constant factor. In particular $f_{i,{\mathbb {Z}}} = c_i \cdot f_{i,{\mathbb {Q}}}$ is satisfied for the final results $f_{i,{\mathbb {Z}}}$ and $f_{i,{\mathbb {Q}}}$ of the two algorithms for suitable $c_i \in {\mathbb {Q}}$. In this way, we show that the outcome of the integer variant of Yun’s algorithm directly produces the square-free factorization $f = c \cdot f_{1,{\mathbb {Z}}}^1 \ldots \cdot f_{n,{\mathbb {Z}}}^n$ from above, so there even is no demand to post-process the result. The combination of the integer version of Yun’s algorithm together with the heuristic of Sect. 3 is then used to assemble the function  .

### Theorem 2

(Yun Factorization and Square-Free Heuristic) 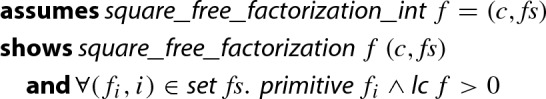


## Square-Free Polynomials in $\mathrm {GF}(p)$

Step 5 in Algorithm 1 mentions the selection of a *suitable* prime *p*, where two conditions have to be satisfied: First, *p* must be coprime to the leading coefficient of the input polynomial *f*. Second, *f* must be square-free in $\mathrm {GF}(p)$, required for Berlekamp’s algorithm to work. Here, for the second condition we use separability as sufficient criterion to ensure square-freeness.

### Example 2

Continuing Example 1, we need to process the polynomial

$$\begin{aligned} f = 4 + 47x - 2x^2 - 23x^3 + 18x^4 + 10x^5. \end{aligned}$$

Selecting $p = 2$ or $p = 5$ is not admissible since these numbers are not coprime to 10, the leading coefficient of *f*. Also $p = 3$ is not admissible since the GCD of *f* and $f'$ is $2 + x$ in $\mathrm {GF}(3)$. Finally, $p = 7$ is a valid choice since the GCD of *f* and $f'$ is 1 in $\mathrm {GF}(7)$, and 7 and 10 are coprime.

In the formalization we must prove that a suitable prime always exists and provide an algorithm which returns such a prime. Whereas selecting a prime that satisfies the first condition is in principle easy—any prime larger than the leading coefficient will do—it is actually not so easy to formally prove that the second condition is satisfiable. We split the problem of computing a suitable prime into the following steps.

- Prove that if *f* is square-free over the integers, then *f* is separable (and therefore square-free) modulo *p* for every sufficiently large prime *p*.
- Develop a prime number generator which returns the first prime such that *f* is separable modulo *p*.

The prime number generator lazily generates all primes and aborts as soon as the first suitable prime is detected. This is easy to model in Isabelle by defining the generator () via  .

Our formalized proof of the existence of a suitable prime proceeds along the following line. Let *f* be square-free over ${\mathbb {Z}}$. Then *f* is also square-free over ${\mathbb {Q}}$ using Gauss’ Lemma. For fields of characteristic 0, *f* is square-free if and only if *f* is separable. Separability of *f*, i.e., coprimality of *f* and $f'$ is the same as demanding that the resultant is non-zero, so we get $\mathsf {res}_{}(f,f') \ne 0$. The advantage of using resultants is that they admit the following property: if *p* is larger than $\mathsf {res}_{}(f,f')$ and the leading coefficients of *f* and $f'$, then $\mathsf {res}_{p}(f,f')\ne 0$, where $\mathsf {res}_{p}(f,g)$ denotes the resultant of *f* and *g* computed in $\mathrm {GF}(p)$. Now we go back from resultants to coprimality, and obtain that *f* and $f'$ are coprime in $\mathrm {GF}(p)$, i.e., *f* is separable modulo *p*.

Whereas the reasoning above shows that any prime larger than $\mathsf {res}_{}(f,f')$,  and  is admitted, we still prefer to search for a small prime *p* since Berlekamp’s algorithm has a worst case lower bound of  operations. The formal statement follows:

### Lemma 15

(Suitable prime) 
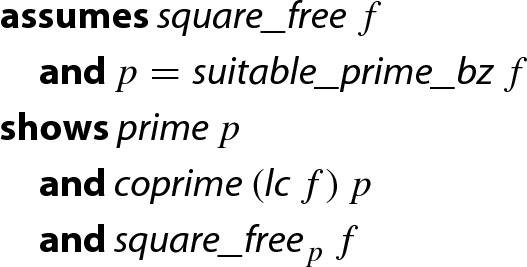


## Berlekamp’s Algorithm

In this section we will describe step 6 of Algorithm 1, i.e., our verified implementation of Berlekamp’s Algorithm to factor square-free polynomials in $\mathrm {GF}(p)$.

### Informal Description

Algorithm 2 briefly describes Berlekamp’s algorithm [5]. It focuses on the core computations that have to be performed. For a discussion on why these steps are performed we refer to Knuth [18, Sect. 4.6.2].
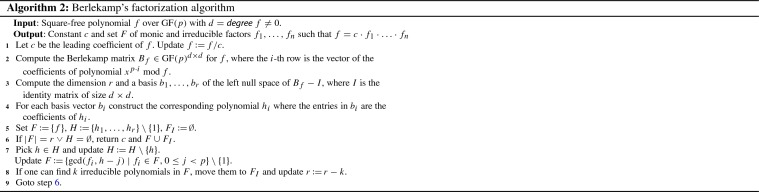


We illustrate the algorithm by continuing Example 2.

#### Example 3

In Algorithm 1, step 6, we have to factor *f* in $\mathrm {GF}(7)[x]$. To this end, we first simplify *f* by

$$\begin{aligned} f \equiv 4 + 5x + 5x^2 + 5x^3 + 4x^4 + 3x^5 \quad (mod 7) \end{aligned}$$

before passing it to Berlekamp’s algorithm.

Step 1 now divides this polynomial by its leading coefficient $c = 3$ in $\mathrm {GF}(7)$ and obtains the new $f:= 6 + 4x + 4x^2 + 4x^3 + 6x^4 + x^5$.

Step 2 computes the Berlekamp matrix as

$$\begin{aligned} B_f = \left( \begin{array}{ccccc} 1 &{}\quad 0 &{}\quad 0 &{}\quad 0 &{}\quad 0 \\ 4 &{} \quad 6 &{}\quad 2 &{}\quad 4 &{} \quad 3 \\ 2 &{}\quad 3 &{}\quad 6 &{}\quad 1 &{}\quad 4 \\ 6 &{}\quad 3 &{}\quad 5 &{}\quad 3 &{}\quad 1 \\ 1 &{}\quad 5 &{}\quad 5 &{}\quad 6 &{}\quad 6 \end{array}\right) \end{aligned}$$

since

$$\begin{aligned} \begin{array}{rcll} x^0 \mathbin {mod }f &{} \equiv &{} 1 &{}(mod 7) \\ x^7 \mathbin {mod }f &{} \equiv &{} 4 + 6x + 2x^2 + 4x^3 + 3x^4 &{}(mod 7) \\ x^{14} \mathbin {mod }f &{} \equiv &{} 2 + 3x + 6x^2 + x^3 + 4x^4 &{}(mod 7) \\ x^{21} \mathbin {mod }f &{} \equiv &{} 6 + 3x + 5x^2 + 3x^3 + x^4 &{}(mod 7) \\ x^{28} \mathbin {mod }f &{} \equiv &{} 1 + 5x + 5x^2 + 6x^3 + 6x^4 &{}(mod 7). \end{array} \end{aligned}$$

Step 3 computes a basis of the left null space of $B_f - I$, e.g., by applying the Gauss–Jordan elimination to its transpose $(B_f -I)^\mathrm {T}$:

$$\begin{aligned} \left( \begin{matrix} 0&{}\quad 0&{}\quad 0&{}\quad 0&{}\quad 0\\ 4&{}\quad 5&{}\quad 2&{}\quad 4&{}\quad 3\\ 2&{}\quad 3&{}\quad 5&{}\quad 1&{}\quad 4\\ 6&{}\quad 3&{}\quad 5&{}\quad 2&{}\quad 1\\ 1&{}\quad 5&{}\quad 5&{}\quad 6&{}\quad 5 \end{matrix}\right) ^{\!\!\mathrm {T}} = \left( \begin{array}{ccccc} 0 &{}\quad 4 &{}\quad 2 &{}\quad 6 &{}\quad 1 \\ 0 &{}\quad 5 &{}\quad 3 &{}\quad 3 &{}\quad 5 \\ 0 &{}\quad 2 &{}\quad 5 &{}\quad 5 &{}\quad 5 \\ 0 &{}\quad 4 &{}\quad 1 &{}\quad 2 &{}\quad 6 \\ 0 &{}\quad 3 &{}\quad 4 &{}\quad 1 &{}\quad 5 \end{array}\right) \hookrightarrow \left( \begin{array}{ccccc} 0 &{}\quad 1 &{}\quad 0 &{}\quad 0 &{}\quad 2 \\ 0 &{}\quad 0 &{}\quad 1 &{}\quad 0 &{}\quad 1 \\ 0 &{}\quad 0 &{}\quad 0 &{}\quad 1 &{}\quad 2 \\ 0 &{}\quad 0 &{}\quad 0 &{}\quad 0 &{}\quad 0 \\ 0 &{}\quad 0 &{}\quad 0 &{}\quad 0 &{}\quad 0 \end{array}\right) \end{aligned}$$

We determine $r = 2$, and extract the basis vectors $b_1 = (1\ 0\ 0\ 0\ 0)$ and $b_2 = (0\ 5\ 6\ 5\ 1)$. Step 4 converts them into the polynomials $h_1 = 1$ and $h_2 = 5x + 6x^2 + 5x^3 + x^4$, and step 5 initializes $H = \{h_2\}$, $F = \{f\}$, and $F_I = \emptyset$.

The termination condition in step 6 does not hold. So in step 7 we pick $h = h_2$ and compute the required GCD s.

$$\begin{aligned} \gcd (f, h_2 - 1)&= 6 + 5x + 6x^2 + 5x^3 + x^4 =: f_1\\ \gcd (f, h_2 - 4)&= 1 + x =: f_2\\ \gcd (f, h_2 - i)&= 1 \qquad \qquad \text {for all }i \in \{0,2,3,5,6\} \end{aligned}$$

Afterwards, we update $F := \{f_1,f_2\}$ and $H := \emptyset$.

Step 8 is just an optimization. For instance, in our implementation we move all linear polynomials from *F* into $F_I$, so that in consecutive iterations they do not have to be tested for further splitting in step 7. Hence, step 8 updates $F_I := \{f_2\}$, $F := \{f_1\}$, and $r := 1$.

Now we go back to step 6, where both termination criteria fire at the same time ($|F| = 1 = r \wedge H = \emptyset$). We return $c \cdot f_1 \cdot f_2$ as final factorization, i.e.,

$$\begin{aligned} f \equiv 3 \cdot (1 + x) \cdot (6 + 5x + 6x^2 + 5x^3 + x^4) \quad (mod 7) \end{aligned}$$

All of the arithmetic operations in Algorithm 2 have to be performed in the prime field $\mathrm {GF}(p)$. Hence, in order to implement Berlekamp’s algorithm, we basically need the following operations: arithmetic in $\mathrm {GF}(p)$, polynomials over $\mathrm {GF}(p)$, the Gauss–Jordan elimination over $\mathrm {GF}(p)$, and GCD-computation for polynomials over $\mathrm {GF}(p)$.

### Soundness of Berlekamp’s Algorithm

Our soundness proof for Berlekamp’s algorithm is mostly based on the description in Knuth’s book.

We first formalize the equations (7, 8, 9, 10, 13, 14) in the textbook [18, p. 440 and 441]. To this end, we also adapt existing proofs from the Isabelle distribution and the AFP; for instance, to derive  (7) in the textbook, we adapted a formalization of the Chinese remainder theorem, which we could find only for *integers* and *naturals*, to be applicable to *polynomials* over fields. For another example, (13) uses the equality $(f + g)^p = f^p + g^p$ where *f* and *g* are polynomials over $\mathrm {GF}(p)$, which we prove using some properties about binomial coefficients that were missing in the library. Having proved these equations, we eventually show that after step 3 of Algorithm 2, we have a basis $b_1,\dots ,b_r$ of the left null space of $B_f - I$.

Now, step 4 transforms the basis into polynomials. We define an isomorphism between the left null space of $B_f - I$ and the *Berlekamp subspace*so that the isomorphism transforms the basis $b_1,\dots ,b_r$ into a *Berlekamp basis*$H_b := \{h_1,\dots ,h_r\}$, a basis of $W_f$. Then we prove that every factorization of *f* has at most *r* factors.

In this proof we do not follow Knuth’s arguments, but formalize our own version of the proof to reuse some results which we have already proved in the development. Our proof is based on another isomorphism between the vector spaces $W_f$ and $\mathrm {GF}(p)^r$ as well as the use of the Chinese remainder theorem over polynomials and the uniqueness of the solution.

#### Lemma 16

Every factorization of a square-free monic polynomial $f \in \mathrm {GF}(p)[x]$ has at most $\dim W_f$ factors.

#### Proof

Let $f \equiv f_1 \cdot \ldots \cdot f_r \quad (mod p)$ be a monic irreducible factorization in $\mathrm {GF}(p)[x]$, which exists and is unique up to permutation since $\mathrm {GF}(p)[x]$ is a unique factorization domain. We show that there exists an isomorphism between the vector spaces $W_f$ and $\mathrm {GF}(p)^r$. Then they have the same dimension and thus every factorization of *f* has at most $\dim W_f = \dim \mathrm {GF}(p)^r = r$ factors, which is the desired result.

First, the following equation holds for any polynomial $g \in W_f$. It corresponds to equation (10) in the textbook [18, p. 440].

10 $$\begin{aligned} g^p - g\ = \prod _{a \in \mathrm {GF}(p)} (g - a). \end{aligned}$$

From this we infer that each $f_i$ divides $\prod _{a \in \mathrm {GF}(p)}(g - a)$. Since $f_i$ is irreducible, $f_i$ divides $g-a$ for some $a \in \mathrm {GF}(p)$ and thus, $(g \mathbin {mod }f_i) = -a$ is a constant.

Now we define the desired isomorphism $\phi$ between $W_f$ and $\mathrm {GF}(p)^r$ as follows:

$$\begin{aligned} \phi :&W_f \rightarrow \mathrm {GF}(p)^r\\&g \mapsto (g \mathbin {mod }f_1, \dots , g \mathbin {mod }f_r) \end{aligned}$$

To show that $\phi$ is an isomorphism, we start with proving that $\phi$ is injective. Let us assume that $\phi \, g = 0$ for some $g \in W_f$. It is easy to show  and $\forall i<r.\ g \equiv \phi \, g \quad (mod f_i)$. Since $v = 0 \in W_f$ satisfies these properties, the uniqueness result of the Chinese remainder theorem guarantees that $g = 0$. This implies the injectivity of $\phi$, since any linear map is injective if and only if its kernel is {0} [2, Proposition 3.2].

To show that $\phi$ is surjective, consider an arbitrary $x = (x_1, \dots , x_r) \in \mathrm {GF}(p)^r$. We show that there exists a polynomial $g \in W_f$ such that $\phi \, g = x$. The Chinese remainder theorem guarantees that there exists a polynomial *g* such that:3

4 $$\begin{aligned}&\forall i<r.\ g \equiv x_i \quad (mod f_i) \end{aligned}$$

Then, for each $i < r$ we have , and so $g^{p} \equiv g \quad (mod f_i)$. Since each $f_i$ is irreducible and *f* is square-free, we have $g^p \equiv g \quad (mod \prod f_i)$. As $\prod f_i = f$, we conclude $g \in W_f$. Finally, $\phi \, g = x$ follows from (4) and the fact that $g \mathbin {mod }f_i$ is a constant. $\square$

As expected, the proof in Isabelle requires more details and it takes us about 300 lines (excluding any previous necessary result and the proof of the Chinese remainder theorem). We define a function for indexing the factors, we prove that both $W_f$ and $\mathrm {GF}(p)^r$ are finite-dimensional vector spaces and also that $\phi$ is a linear map. Since each equation of the proof involves polynomials over $\mathrm {GF}(p)$ (so everything is modulo *p*), we also proved facts like  and so on. In addition, we also extend an existing AFP entry [22] about vector spaces for some necessary results about linear maps, isomorphisms between vector spaces, dimensions, and bases.

Once having proved that $H_b$ is a Berlekamp basis for *f* and that the number of irreducible factors is $|H_b|$, we prove (14); for every divisor $f_i$ of *f* and every $h \in H_b$, we have

14 $$\begin{aligned} f_i = \prod _{0 \le j < p} \gcd (f_i, h - j). \end{aligned}$$

Finally, it follows that every non-constant reducible divisor $f_i$ of *f* can be properly factored by $\gcd (f_i, h - j)$ for suitable $h \in H_b$ and $0 \le j < p$.

In order to prove the soundness of steps 5–9 in Algorithm 2, we use the following invariants—these are not stated by Knuth as equations. Here, $H_{old }$ represents the set of already processed polynomials of $H_b$.

1. $$f = \prod (F \cup F_I)$$.
2. All $f_i \in F \cup F_I$ are monic and non-constant.
3. All $f_i \in F_I$ are irreducible.
4. $$H_b = H \cup H_{old }$$.
5. $\gcd (f_i,h - j) \in \{1,f_i\}$ for all $h \in H_{old }$, $0 \le j < p$ and $f_i \in F \cup F_I$.
6. $$|F_I| + r = |H_b|$$.

It is easy to see that all invariants are initially established in step 5 by picking $H_{old } = \{1\} \cap H_b$. In particular, invariant 5 is satisfied since the GCD of the monic polynomial *f* and a constant polynomial *c* is either 1 (if $c\ne 0$) or *f* (if $c=0$).

It is also not hard to see that step 7 preserves the invariants. In particular, invariant 5 is satisfied for elements in $F_I$ since these are irreducible. Invariant 1 follows from (14).

The irreducibility of the final factors that are returned in step 6 can be argued as follows. If $|F| = r$, then by invariant 6 we know that $|H_b| = |F \cup F_I|$, i.e., $F \cup F_I$ is a factorization of *f* with the maximum number of factors, and thus every factor is irreducible. In the other case, $H = \emptyset$ and hence $H_{old } = H_b$ by invariant 4. Combining this with invariant 5 shows that every element $f_i$ in $F \cup F_I$ cannot be factored by $\gcd (f_i,h - j)$ for any $h \in H_b$ and $0 \le j < p$. Since $H_b$ is a Berlekamp basis, this means that $f_i$ must be irreducible.

Putting everything together we arrive at the formalized main soundness statement of Berlekamp’s algorithm. As in Sect. 6.3 we will integrate the distinct-degree factorization [18, p. 447 and 448], the algorithm takes, besides the monic polynomial *f* to be factored, an extra argument $d \in {\mathbb {N}}$ such that any degree-*d* factor of *f* is known to be irreducible. Fixing $d=1$ yields the usual Berlekamp’s algorithm. The final statement looks as follows.

#### Theorem 3

(Berlekamp’s Algorithm for monic polynomials) 
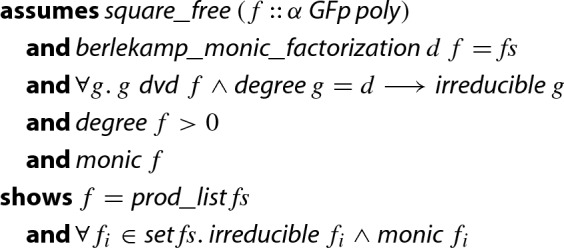


In order to prove the validity of the output factorization, we basically use the invariants mentioned before. However, it still requires some tedious reasoning.

### Formalizing the Distinct-Degree Factorization Algorithm

The distinct-degree factorization (cf. [18, p. 447 and 448]) is an algorithm that splits a square-free polynomial into (possibly reducible) factors, where irreducible factors of each factor have the same degree. It is commonly used before applying randomized algorithms to factor polynomials, and can also be used as a preprocessing step before Berlekamp’s algorithm. Algorithm 3 briefly describes how it works.
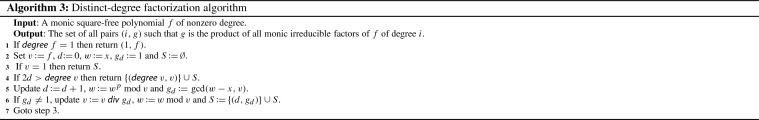


We implement the algorithm in Isabelle/HOL as  . Termination follows from the fact that difference between *d* and the degree of *v* decreases in every iteration. The key to the soundness of the algorithm is the fact that any irreducible polynomial *f* of degree *d* divides the polynomial $x^{p^d} - x$ and does not divide $x^{p^c}-x$ for $1 \le c < d$. The corresponding Isabelle’s statement looks as follows where the polynomial *x* is encoded as  , i.e., $1 \cdot x^1$.

#### Lemma 17


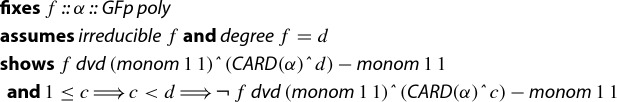


Knuth presents such a property as a consequence of an exercise in his book, whose proof is sketched in prose in just 5 lines [18, Exercise 4.6.2.16]. In comparison, our Isabelle proof required more effort: it took us about 730 lines, above all because we proved several facts and subproblems:^6^

- Given a degree-*n* irreducible polynomial $f \in \mathrm {GF}(p)[x]$, the $p^n$ polynomials of degree less than *n* form a field under arithmetic modulo *f* and *p*.
- Any field with $p^n$ elements has a generator element $\xi$ such that the elements of the field are $\{0,1,\xi , \xi ^2, \dots , \xi ^{p^n-2}\}$. We do not follow Knuth’s short argument in this step, but we reuse some theorems of the Isabelle library to provide a proof based on the existence of an element in the multiplicative group of the finite field with the adequate order.
- Given a degree-*n* irreducible polynomial $f \in \mathrm {GF}(p)[x]$, $x^{p^m} - x$ is divisible by *f* if and only if *m* is a multiple of *n*. Essentially, we are proving that $\mathrm {GF}(p^n)$ is a subfield of $\mathrm {GF}(p^m)$ if and only if *n* divides *m*.

The difference between the sizes of Knuth’s and our proofs is also due to some properties which Knuth leaves as exercises. For instance, we show that $a^{p^n} = a$ for any element $a\in \mathrm {GF}(p)$, also that $(f+g)^{p^n} = f^{p^n} + g^{p^n}$ in the ring $\mathrm {GF}(p)[x]$, for natural numbers $x>1$, $a>0$ and $b>0$ we demonstrate  and some other properties like these ones which cause the increase in the number of employed lines. The whole formalization of these facts, the termination-proof of the algorithm and its soundness can be seen in the file Distinct_Degree_Factorization.thy of our development.

Once we have the distinct-degree factorization formalized, it remains to find a way to split each factor that we have found into the desired irreducible factors, but this can just be done by means of the Berlekamp’s algorithm. This way, we have two ways of factoring polynomials in $\mathrm {GF}(p)[x]$:

- Using Berlekamp’s algorithm directly.
- Preprocessing the polynomial using the distinct-degree factorization and then apply Berlekamp’s algorithm to the factors.

We verified both variants as a single function  where a Boolean constant is used to enable or disable the preprocessing via distinct-degree factorization. Our experiments revealed that currently the preprocessing slows down the factorization algorithm, so the value of the Boolean constant is set to disable the preprocessing. However, since distinct degree factorization heavily depends on polynomial multiplication, the preprocessing might pay off, once more efficient polynomial multiplication algorithms become available in Isabelle.

Independent of the value of the Boolean constant, the final type-based statement for the soundness of  is as follows.

#### Theorem 4

(Finite Field Factorization) 
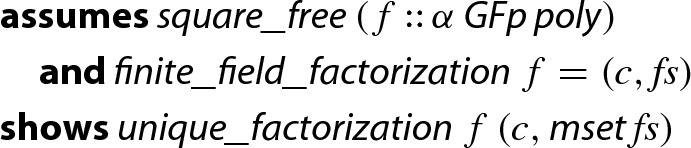


Here,  converts a list into a multiset, and  demands that the given factorization is the unique factorization of *f*, i.e., *c* is the leading coefficient of *f* and $fs$ a list of irreducible and monic factors such that $f = c \cdot {\prod \, fs }$. Uniqueness follows from the general theorem that the polynomials over fields form a unique factorization domain.

### Implementing Finite Field Factorization

The soundness of Theorem 4 is formulated in a *type-based setting*. In particular, the function  has typeIn our use case, recall that Algorithm 1 first computes a prime number *p*, and then invokes a factorization algorithm (such as Berlekamp’s algorithm) on $\mathrm {GF}(p)$. This requires Algorithm 1 to construct a new type $\tau$ with  depending on the value of *p*, and then invoke  for type .

Unfortunately, this is not possible in Isabelle/HOL. Hence, Algorithm 1 requires a finite field factorization algorithm to have a type likewhere the first argument is the dynamically chosen prime *p*.

The final goal is to prove Theorem 4 but just involving integers, integer polynomials and integer lists, and then avoiding statements and definitions that require anything of type  (or in general, anything involving the type ).

The solution is to follow the steps already detailed in Sect. 3. We briefly recall the main steps here:

- We implement a record-based copy of all necessary algorithms like Gauss–Jordan elimination,  and  where the type-based arithmetic operations are replaced by operations in the record.
- In a locale that assumes a sound implementation of the record-based arithmetic and that fixes *p* such that , we develop transfer rules to relate the new implementation of all subalgorithms that are invoked with the corresponding type-based algorithms.
- Out of the locale, we define a function  which dynamically selects an efficient implementation of $\mathrm {GF}(p)$ depending on *p*, by means of  . This function has the desired type. Its soundness statement can be proven by means of the transfer rules, but the resulting theorem still requires that .
- Thanks to local type definitions, such a premise is replaced by .

As the approach is the same as the presented in Sect. 3, we omit here the details. We simply remark that the diagnostic commands  and  were helpful to see why certain transfer rules could initially not be proved automatically; these commands nicely pointed to missing transfer rules.

Most of the transfer rules for non-recursive algorithms were proved mainly by unfolding the definitions and finishing the proof by . For recursive algorithms, we often perform induction via the algorithm. To handle an inductive case, we locally declare transfer rules (obtained from the induction hypothesis), unfold one function application iteration, and then finish the proof by .

Still, problems arose in case of underspecification. For instance it is impossible to prove an unconditional transfer rule for the function  that returns the head of a list using the standard relator for lists, ; when the lists of type  and  are empty, we have to relate  with . To circumvent this problem, we had to reprove invariants that  is invoked only on non-empty lists.

Similar problems arose when using matrix indices where transfer rules between matrix entries $A_{ij}$ and $B_{ij}$ are available only if *i* and *j* are within the matrix dimensions. So, again we had to reprove the invariants on valid indices—just unfolding the definition and invoking  was not sufficient.

Although there is some overhead in this approach—namely by copying the type-based algorithms into record-based ones, and by proving the transfer rules for each of the algorithms—it still simplifies the overall development: once this setup has been established, we can easily transfer statements about properties of the algorithms, without having to copy or adjust their proofs.

This way, we obtain a formalized and executable factorization algorithm for polynomials in finite fields where the prime number *p* can be determined at runtime, and where the arithmetic in $\mathrm {GF}(p)$ is selected dynamically without the risk of integer overflow. The final theorem follows, which is the integer-based version of Theorem 4.

#### Theorem 5

(Finite Field Factorization on Integers)  


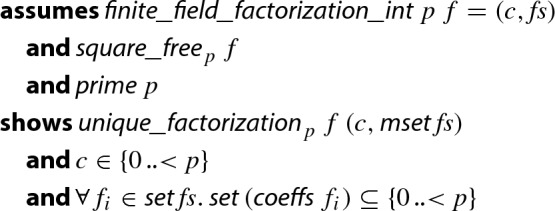


In summary, the development of the separate implementation is some annoying overhead, but still a workable solution. In numbers: Theorem 4 requires around 4300 lines of difficult proofs whereas Theorem 5 demands around 600 lines of easy proofs.

## Mignotte’s Factor Bound

Reconstructing the polynomials proceeds by obtaining factors modulo $p^k$. The value of *k* should be large enough, so that any coefficient of any factor of the original integer polynomial can be determined from the corresponding coefficients in ${\mathbb {Z}}/{p^k}{\mathbb {Z}}$. We can find such *k* by finding a bound on the coefficients of the factors of *f*, i.e., a function  such that the following statement holds:

### Lemma 18

(Factor Bound) 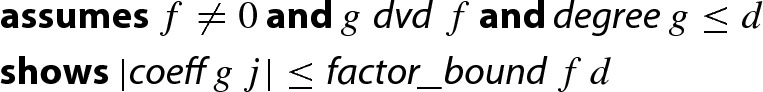


Clearly, if *b* is a bound on the absolute value of the coefficients, and $p^k > 2\cdot b$ then we can encode all required coefficients: In ${\mathbb {Z}}/{p^k}{\mathbb {Z}}$ we can represent the numbers $\{-\lfloor \frac{p^k-1}{2}\rfloor ,\dots ,{\lceil }{\frac{p^k-1}{2}}{\rceil }\} \supseteq \{-b,\dots ,b\}$.

The *Mignotte bound* [27] provides a bound on the absolute values of the coefficients. The Mignotte bound is obtained by relating the *Mahler measure* of a polynomial to its coefficients. The Mahler measure is defined as follows:where  and $r_1, \ldots , r_n$ are the complex roots of *f* taking multiplicity into account. For nonzero *f*,  is a nonzero integer. It follows that . The equality  easily follows by the definition of the Mahler measure. We conclude that  if *g* is a factor of *f*.

The Mahler measure is bounded by the coefficients from above through Landau’s inequality:Mignotte showed that the coefficients also bound the measure from below:  whenever . Putting this together we get:Consequently, we could define  as follows:Such a definition of  was the one used in our previous work [10]. However, we have introduced an important improvement at this point to get tighter factor bounds by means of integrating Graeffe transformations.

Given a complex polynomial $f = c \prod _i (x-r_i)$, we can define its *m*-th Graeffe transformation as the polynomial $f_m = c^{2^m} \prod _{i} (f-r_i^{2^m})$.

These polynomials are easy to compute, since

5 $$\begin{aligned} f_m={\left\{ \begin{array}{ll} f, &{} \text {if }m=0.\\ c \cdot (g^2-xh^2), &{} \text {otherwise}\\ \end{array}\right. } \end{aligned}$$

where *g* and *h* are the polynomials that separates $f_{m-1}$ into its even and odd parts such that $f_{m-1}(x) = g(x^2)+xh(x^2)$. For instance, if $f_{m-1} = ax^4+bx^3+cx^2+dx+e$ then $g=ax^2 + cx + e$ and $h = bx+d$.

We implement both the definition of Graeffe transformation and (5) and then we show they are equivalent. The former one makes proofs easier, whereas the latter one is devoted for computational purposes and thus used during code generation. At this point we introduce functions involving lists, e.g.  (to obtain the odd and even parts of a polynomial) and  (to split a list into another two ones in which elements are alternated). For a polynomial *f* of degree *n*, we then prove three important facts:

The first one follows from the definition of Mahler measure and Graeffe transformation, the second one follows from the first property and the Landau’s inequality and the third one is obtained from the definition of Mahler measure and the Mignotte’s inequality.

The implementation of an approximation for the Mahler measure based on Graeffe transformations requires the computation of *n*-th roots, which already can be done thanks to previous work based on the Babylonian method [30]. That work implements functions to decide whether $\root n \of {a} \in {\mathbb {Q}}$ and compute the ceiling and floor of $\root n \of {a}$. The computation of the *n*-th root of a number is based on a variant of Newton iteration, but involving integer divisions instead of floating point or rational divisions, i.e., each occurrence of  in the algorithm has been substituted by . We must also choose a starting value in the iteration, which must be larger than the *n*-th root. This property is essential, since the algorithm will abort as soon as we fall below the *n*-th root. Thus, the starting value is defined as $2 ^ {\lceil \lceil \log _2 {a}\rceil / n\rceil }$.

This of course requires a function to approximate logarithms. At first, the development [30] implemented this approximation in a naive way, i.e., multiplying the base until we exceed the argument, which causes an impact on the efficiency and avoid an improvement on the performance if Graeffe transformations are integrated.

To tackle this, we implement the discrete logarithm function in a manner similar to a repeated squaring exponentiation algorithm. This way, we get a fast logarithm algorithm, as required for Graeffe transformations. This algorithm allows us to derive the floor- and ceiling-logarithm functions. We also connect them to the  function working on real numbers.

### Lemma 19


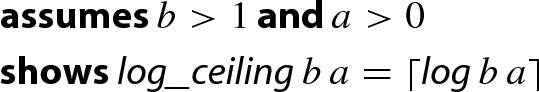


Once we have a fast logarithm algorithm implemented, we can now define a function  which returns an upper bound for the Mahler measure, based on the Graeffe transformations. We refer to the sources and [9] for the details of the implementation. The function receives three parameters: the number *m* of Graeffe transformations which are performed, a scalar *c* and the polynomial *f*. Using the previous properties, we can now prove the following important fact:Putting all together, for a polynomial *g* of  we have:Consequently, we can define  based on , but firstly it remains to decide the number of iterations (the value of *m*), in a balance between the precision of the bound and the computational time needed to get it. First we tried too high numbers which gave good results for small polynomials but have been too expensive to compute for larger polynomials, i.e., where the factor-bound computation resulted in a timeout. After some experiments we finally selected a value of $m = 2$ and defined  in Isabelle as follows, which is a function that satisfies the statement presented at the beginning of this section:
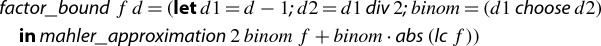


For $m = 2$ we get quite some decrease in the estimation of the Mahler measure. Let us show two examples of it. Consider the polynomials $f = x^8+ 8x^7+ 47x^6+ 136x^5+ 285x^4+ 171x^3-20x^2-21x+ 2$ and $g=2x^8-16x^7+ 26x^6-10x^5-41x^4+ 89x^3-87x^2+ 52x-10$ that appear in [1, Sects. 3.6.1 and 3.6.2].

The paper estimates a Mahler measure of 197 for *f* and 33.4 for *g*, Our results are presented in Table 1. They clearly illustrate an improved precision when applying Graeffe’s transformation a few times.

Interestingly, even with the slightly worse estimation of 200 for *f* when $m=2$, we result in better factor bounds: they report 1181 and 200 for the largest coefficient for a factor of degree 4 of *f* and *g*, respectively, whereas our  results in 604 and .

**Table 1:** Approximating the Mahler measure of the polynomials *f* and *g*

*m*		
0	363	144
1	221	38
2	200	33
3	196	33
4	196	32

So in both cases, the Mahler measure estimations are close to the ones in [1] (with $m = 2$), but we manage to get much smaller coefficient bounds via the Mignotte bound (roughly a factor of 2).

In order to compute a factor bound via Theorem 18 it remains to choose a bound *d* on the degrees of factors of *f* that we require for reconstruction. A simple choice is , but we can do slightly better. After having computed the Berlekamp factorization, we know the degrees of the factors of *f* in $\mathrm {GF}(p)$. Since the degrees will not be changed by the Hensel lifting, we also know the degrees of the polynomials $h_i$ in step 8 of Algorithm 1.

Since in step 9 of Algorithm 1 we will combine at most half of the factors, it suffices to take , where we assume that the sequence $h_1,\ldots ,h_m$ is sorted by degree, starting with the smallest. In the formalization this gives rise to the following definition:Note also that in the reconstruction step we actually compute factors of . Thus, we have to multiply the factor bound for *f* by .

### Example 4

At the end of Example 3 we have the factorization $f = 4 + 47x - 2x^2 - 23x^3 + 18x^4 + 10x^5 \equiv 3 \cdot (1 + x) \cdot (6 + 5x + 6x^2 + 5x^3 + x^4) \quad (mod 7)$.

We compute . With the bound used in our previous work [10], we have to be able to represent coefficients of at most $$10 \cdot \lfloor \sqrt{{\left( {\begin{array}{c}4\\ 2\end{array}}\right) }^2 \cdot (4^2 + 47^2 + 2^2 + 23^2 + 18^2 + 10^2)}\rfloor = 3380$$, i.e., the numbers $\{-3380, \ldots , 3380\}$. In contrast, using the new estimations we can reduce the bound, and compute that it suffices to represent coefficients of at most 1730. Thus the modulus has to be larger than $2 \cdot 1730 = 3460$. Hence, in step 7 of Algorithm 1 we choose $k = 5$, since this is the least number *k* such that $p^k = 7^k > 3460$.

Finally, we report that our previous oracle implementation [31, Sect. 4] had a flaw in the computation of a suitable degree bound *d*, since it just defined *d* to be the half of the degree of *f*. This choice might be insufficient:^7^ Consider the list of degree of the $h_i$ to be [1, 1, 1, 1, 1, 5]. Then the product $h_1 \cdot h_6$ of degree 6 might be a factor of *f*, but the degree bound in the old implementation was computed as $\frac{1+1+1+1+1+5}{2} = 5$, excluding this product. This wrong choice of *d* was detected only after starting to formalize the required degree bound.

## Hensel Lifting

Given a factorization in $\mathrm {GF}(p)[x]$:which Berlekamp’s algorithm provides, the task of the Hensel lifting is to compute a factorization in ${\mathbb {Z}}/{p^k}{\mathbb {Z}} [x]$Hensel’s lemma, following Miola and Yun [28], is stated as follows.

### Lemma 20

(Hensel) Consider polynomials *f* over ${\mathbb {Z}}$, $g_1$ and $h_1$ over $\mathrm {GF}(p)$ for a prime *p*, such that $g_1$ is monic and $f \equiv g_1\cdot h_1 \quad (mod p)$. For any $k \ge 1$, there exist polynomials $g_k$ and $h_k$ over ${\mathbb {Z}}/{p^k}{\mathbb {Z}}$ such that $g_k$ is monic, $f \equiv g_k \cdot h_k\, (mod p^k)$, $g_k \equiv g_1 \quad (mod p)$, $h_k \equiv h_1 \quad (mod p)$. Moreover, if *f* is monic, then $g_k$ and $h_k$ are unique (mod ${p^k}$).

The lemma is proved inductively on *k* where there is a one step lifting from ${\mathbb {Z}}/{p^k}{\mathbb {Z}}$ to ${\mathbb {Z}}/{p^{k+1}}{\mathbb {Z}}$. To be more precise, the one step lifting assumes polynomials $g_k$ and $h_k$ over ${\mathbb {Z}}/{p^k}{\mathbb {Z}}$ satisfying the conditions, and computes the desired $g_{k+1}$ and $h_{k+1}$ over ${\mathbb {Z}}/{p^{k+1}}{\mathbb {Z}}$.

As explained in Sect. 3, it is preferable to carry on the proof in the type-based setting whenever possible, and indeed we proved the one-step lifting in this way.

### Lemma 21

(Hensel lifting–one step) 
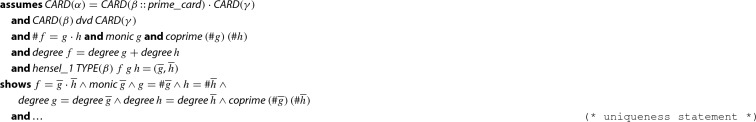


Here,  represents $p^{k+1}$,  represents *p*, and  represents $p^k$. The prefix “$\#$” denotes the function that converts polynomials over integer modulo *m* into those over integer modulo *n*, where the type inference determines *n*.

Unfortunately, we could not see how to use Lemma 21 in the inductive proof of Lemma 20 in a type-based setting. A type-based statement of Lemma 20 would have an assumption like . Then the induction hypothesis would look like6and the goal statement would be . There is no hope to be able to apply the induction hypothesis (6) for this goal, since the assumptions are clearly incompatible. A solution to this problem seems to require extending the induction scheme to admit changing the type variables, and produce an induction hypothesis like  where $?\alpha$ can be instantiated. Unfortunately this is not possible in Isabelle/*HOL*. A rule that seems useful for this problem is the cross-type induction schema [6], which is a general-purpose axiom for cross-type well-founded induction and recursion. However, it is not admissible in current HOL.

We therefore formalized most of the reasoning for Hensel’s lemma on *integer* polynomials in the integer-based setting (cf. Sect. 3.2), so that the modulus (the *k* in the $p^k$) can be easily altered within algorithms and inductive proofs.^8^ The binary version of Hensel’s lemma is formalized as follows, and internally one step of the Hensel lifting is applied over and over again, i.e., the exponents are *p*, $p^2$, $p^3$, $p^4$, ... [28, Sect. 2.2]. In the statement, Isabelle’s syntax $\exists !$ represents the unique existential quantification.

### Lemma 22

(Hensel lifting–multiple steps, binary) 
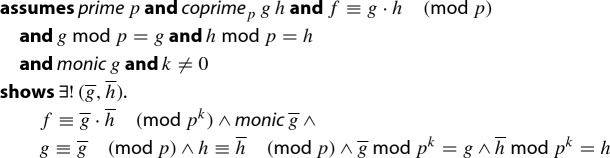


It is also possible to lift in one step from $p^k$ to $p^{2k}$, which is called the *quadratic Hensel lifting*, cf. [28, Sect. 2.3]. In this paper we consider several combinations of one-step and quadratic Hensel lifting.

In the following we use the symbols $\rightarrow$, $\Rightarrow$, and $\searrow$ to indicate a one-step Hensel lifting step, a quadratic Hensel lifting step, and the operation which decreases the modulus from $p^{i+j}$ to $p^i$, respectively. For each alternative, we immediately illustrate the sequence of operations that are performed to produce a factorization modulo $p^{51}$.

1. Repeated one-step lifting:
  $$\begin{aligned} p^1 \rightarrow p^2 \rightarrow p^3 \rightarrow \dots \rightarrow p^{51} \end{aligned}$$
2. Repeated quadratic lifting [28, Sect. 2.3], which applies the quadratic Hensel lifting until $p^{2^\ell } \ge k$ and then finally take remainder operation modulo $p^k$ in order to convert the ${\mathbb {Z}}/{p^{2^\ell }}{\mathbb {Z}}$ factorization into a ${\mathbb {Z}}/{p^k}{\mathbb {Z}}$ factorization. Hence, the operations for $k = 51$ are:
  $$\begin{aligned} p^1 \Rightarrow p^2 \Rightarrow p^4 \Rightarrow p^8 \Rightarrow p^{16} \Rightarrow p^{32} \Rightarrow p^{64} \searrow p^{51} \end{aligned}$$
3. Combination of one-step and quadratic liftings. Lifting to $p^k$ proceeds by recursively computing the lifting to $p^{\lfloor \frac{k}{2} \rfloor }$, then perform a quadratic Hensel lifting to $p^{2 \cdot \lfloor \frac{k}{2} \rfloor }$, and if *k* is odd, do a final linear Hensel lifting to $p^k$. Hence, the operations are:
  $$\begin{aligned} p^1 \Rightarrow p^2 \rightarrow p^3 \Rightarrow p^6 \Rightarrow p^{12} \Rightarrow p^{24} \rightarrow p^{25} \Rightarrow p^{50} \rightarrow p^{51} \end{aligned}$$
  Although the numbers stay smaller than in the second approach, this approach has the disadvantage that the total number of Hensel liftings is larger.
4. Combination of quadratic lifting and modulus decrease. To obtain a lifting for $p^k$, we recursively compute the lifting to $p^{\lceil \frac{k}{2} \rceil }$, then do a quadratic Hensel lifting to $p^{2 \cdot \lceil \frac{k}{2} \rceil }$, and if *k* is odd, do a final decrease operation to $p^k$.
  $$\begin{aligned} p^1 \Rightarrow p^2 \Rightarrow p^4 \Rightarrow p^8 \searrow p^7 \Rightarrow p^{14} \searrow p^{13} \Rightarrow p^{26} \Rightarrow p^{52} \searrow p^{51} \end{aligned}$$
  In comparison to the third approach, we have slightly larger numbers, but we can replace (expensive) one-step Hensel liftings by the cheap modulus decrease.

In our experiments, it turned out that alternative 4 is faster than 2, which in turn is faster than 3. Alternative 2 is faster than 1 in contrast to the result of Miola and Yun [28, Sect. 1].^9^ Therefore, the current formalization adopts alternative 4, whereas our previous version [10] implemented alternative 2.

We further extend the binary (quadratic) lifting algorithm to an *n*-ary lifting algorithm. It inputs a list $$ fs $$ of factors modulo *p* of a square-free polynomial *f*, splits it into two groups $$ fs _1$$ and $$ fs _2$$, then applies the binary Hensel lifting to $$\left( \prod fs _1\right) \cdot \left( \prod fs _2\right) \equiv f \quad (mod p)$$ obtaining $$g_1 \cdot g_2 \equiv f \quad (mod p^k)$$, and finally calls the algorithm recursively to both $$\prod fs _1 \equiv g_1$$ and $$\prod fs _2 \equiv g_2 \quad (mod p)$$.

Since the runtime of the binary Hensel lifting is nonlinear to the degree, the lists $$ fs _1$$ and $$ fs _2$$ should better be balanced so that their products have similar degrees. To this end, we define the following  instead of lists:
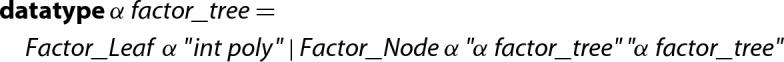


We implement operations involving this datatype, such as obtaining the multiset of factors of a factor tree, subtrees and product of factor trees modulo *p*. This change from lists to trees allows us to implement the multifactor Hensel lifting [33, Chapter 15.5] as well as easily balance the involved trees with respect to the degree, that is, we construct the tree so that the sum of the degrees of the factors of *f* modulo *p* which are stored in the left-branch is similar to the sum of the degrees of the factors stored in the right-branch of the tree. This way, we avoid expensive computations of Hensel lifting steps involving high-degree polynomials. We refer to the 1st edition of the textbook [33] for further details on factor trees and to the Isabelle sources for our implementation.

The final lemma that states the soundness of the Hensel lifting.

### Lemma 23

(Hensel Lifting–general case) 
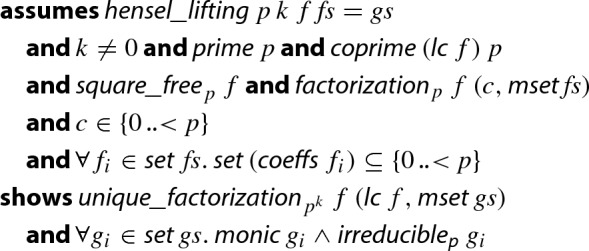


Note that uniqueness follows from the fact that the preconditions already imply that *f* is *uniquely* factored in ${\mathbb {Z}}/{p}{\mathbb {Z}}$—just apply Theorem 5.

We do not go into details of the proofs, but briefly mention that also here local type definitions have been essential. The reason is that the computation relies upon the extended Euclidean algorithm applied on polynomials over $\mathrm {GF}(p)$. Since the soundness theorem of this algorithm is available only in a type-based version in the Isabelle distribution, we first convert it to the integer representation of $\mathrm {GF}(p)$ and a record-based implementation as in Sect. 3.

We end this section by proceeding with the running example, without providing details of the computation.

### Example 5

Applying the Hensel lifting on the factorization of Example 3 with $k = 5$ from Example 4 yields

$$\begin{aligned} f \equiv&\ 3 \cdot (2885 + x) \cdot (14\,027 + 7999x + 13\,691x^2 + 7201x^3 + x^4) \quad (mod p^k) \end{aligned}$$

## Reconstructing True Factors

For formalizing step 9 of Algorithm 1, we basically follow Knuth, who describes the reconstruction algorithm briefly and presents the soundness proof in prose [18, steps F2 and F3, p. 451 and 452]. At this point of the formalization the De Bruijn factor is quite large, i.e., the formalization is by far more detailed than the intuitive description given by Knuth.

The following definition presents (a simplified version of) the main worklist algorithm, which is formalized in Isabelle/HOL via the  command.^10^
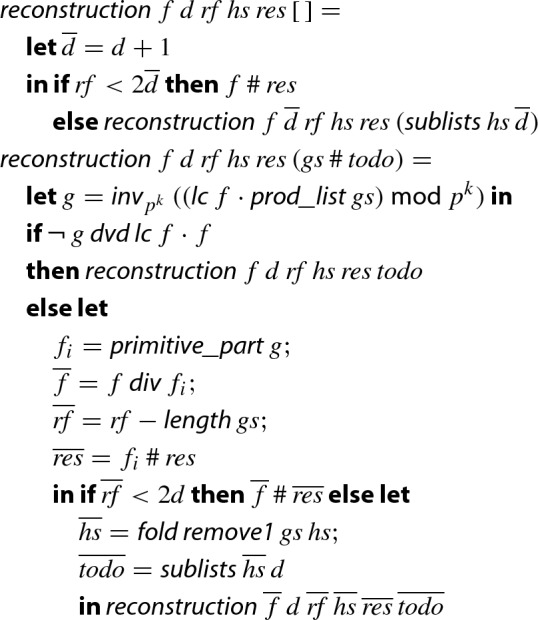


Here, $rf$ is supposed to be the number of remaining factors, i.e., the length of ;  denotes the list of length-*d* sublists of $hs$; and  is the inverse modulo function, which converts a polynomial with coefficients in $\{0,\ldots ,m\}$ into a polynomial with coefficients in $\{-\lfloor \frac{m-1}{2}\rfloor ,\ldots , {\lceil }{\frac{m-1}{2}}{\rceil }\}$, where the latter set is a superset of the range of coefficients of any potential factor of , cf. Sect. 7.

Basically, for every sublist $$ gs $$ of $$ hs $$ we try to divide  by the reconstructed potential factor *g*. If this is possible then we store $$f_i$$, the primitive part of *g*, in the list $$ res $$ of resulting integer polynomial factors and update the polynomial *f* and its factorization $$ hs $$ in $${\mathbb {Z}}/{p^k}{\mathbb {Z}} $$ accordingly. When the worklist becomes empty or a factor is found, we update the number $$ rf $$ of remaining factors $$ hs $$ and the length *d* of the sublists we are interested in. Finally, when we have tested enough sublists ($$ rf < 2d$$) we finish.

For efficiency, the actual formalization employs three improvements over the simplified version presented here.

- Values which are not frequently changed are passed as additional arguments. For instance  is provided via an additional argument and not recomputed in every invocation of .
- For the divisibility test we first test whether the constant term  of the candidate factor *g* divides that of . In our experiments, in over 99% of the cases this simple integer divisibility test can prove that *g* is not a factor of . This test is in particular efficient, since the constant term of *g* is just the product of the constant terms of the polynomials in *gs*, so that one can execute the test without computing *g* itself.
- The enumeration of sublists is made parametric, and we developed an efficient generator of sublists which reuses results from previous iterations. Moreover, the sublist generator also shares computations to calculate the constant term of *g*.

### Example 6

Continuing Example 5, we have only two factors, so it suffices to consider $d = 1$. We obtain the singleton sublists $[g_1] = [2885 + x]$ and $[g_2] = [14 027 + 7999x + 13 691x^2 + 7201x^3 + x^4]$. The constant term of  is the inverse modulo of $(10 \cdot 2885) \mathbin {mod }p^k$, i.e., $-4764$, and similarly, for $g_2$ we obtain 5814. Since neither of them divides 40, the constant term of , the algorithm returns [*f*], i.e., *f* is irreducible.

The formalized soundness proof of  is much more involved than the paper proof; it is proved inductively with several invariants, for instance

- correct input:
- corner cases: $$2d \le rf $$, $$ todo \ne [\,] \longrightarrow d < rf $$, $$d = 0 \longrightarrow todo = [\,]$$
- irreducible result:
- properties of prime: ,
- factorization mod $$p^k$$:
- normalized input:
- factorization over integers: the polynomial $f \cdot \prod \! res$ stays constant throughout the algorithm
- all factors of  with degree at most  have coefficients in the range $\{-\lfloor \frac{p^k-1}{2}\rfloor ,\dots ,{\lceil }{\frac{p^k-1}{2}}{\rceil }\}$
- all non-empty sublists $gs$ of $hs$ of length at most *d* which are not present in $todo$ have already been tested, i.e., these $gs$ do not give rise to a factor of *f*

The hardest parts in the proofs were to ensure the validity of all invariants after a factor *g* has been detected—since then nearly all parameters are changed—and to ensure that the final polynomial *f* is irreducible when the algorithm terminates.

In total, we achieve the following soundness result, which already integrates many of the results from the previous sections. Here,  is a simple composition of the finite field factorization algorithm (that is, the function  which internally uses the Berlekamp factorization) and the Hensel lifting, and  invokes  with the right set of starting parameters.

### Theorem 6

(Zassenhaus Reconstruction of Factors) 
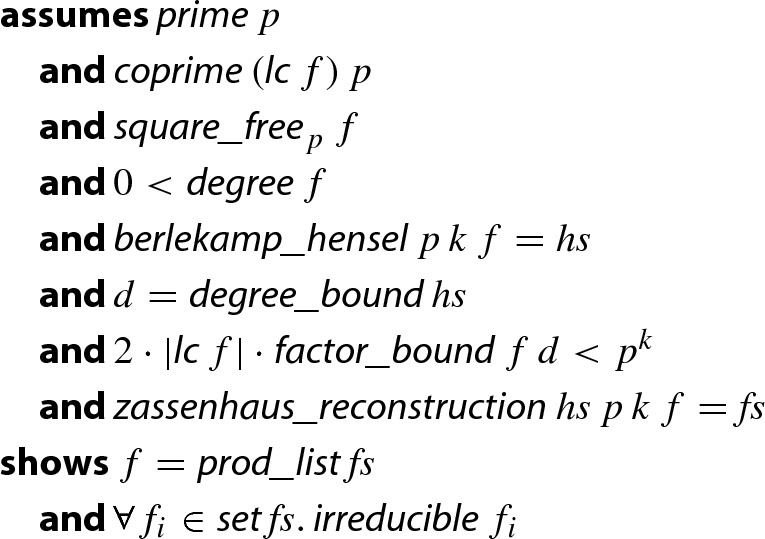


The worst-case runtime of this factor-reconstruction algorithm is known to be exponential. We also have a polynomial-time version based on the lattice reduction algorithm [7, 11], but this contribution goes beyond the scope of this paper.

## Assembled Factorization Algorithm

At this point, it is straightforward to combine the algorithms presented in Sects. 5 to 9 to get a factorization algorithm for square-free polynomials. 
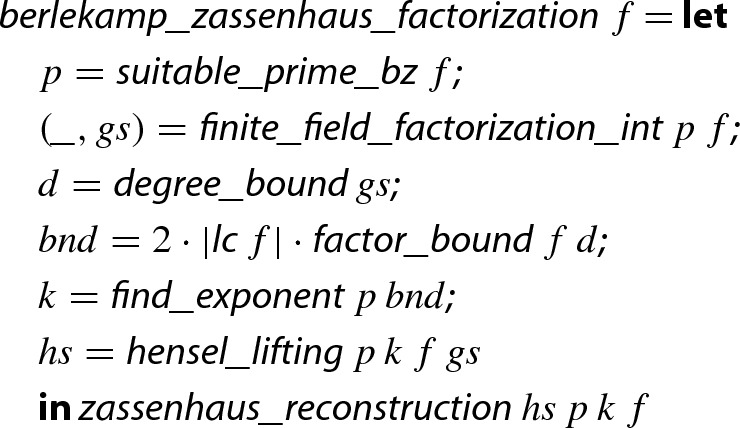
 Here,  just computes an exponent *k* such that $$p^k > bnd $$.

It satisfies the following soundness theorem.

### Theorem 7

(Berlekamp–Zassenhaus Algorithm) 
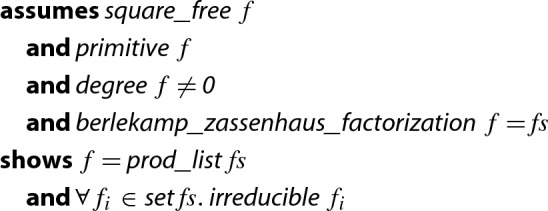


Putting this together with the square-free factorizaton algorithm presented in Sect. 4, we now assemble a factorization algorithm for integer polynomials 
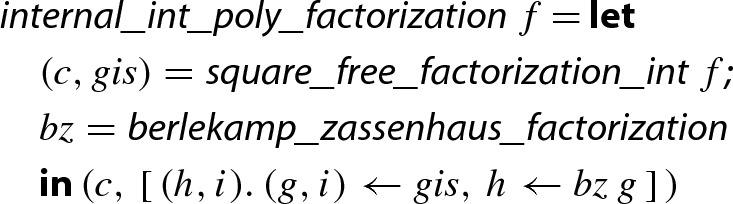
 and prove its soundness:

### Theorem 8

(Factorization of Integer Polynomials) 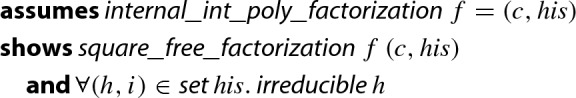


So, we get a factorization algorithm that works for any integer polynomial. But we can do it even better: Performance improves if we include reciprocal polynomials when , since then the values of  and  are swapped, and thus the value of $bnd$ in the definition of  decreases.

The *reciprocal polynomial* of polynomial $f = \sum _{i=0}^n a_ix^i$ is $\sum _{i=0}^n a_{n-i}x^i$, and is defined in Isabelle as . Reciprocal polynomials satisfy some important properties that we have proved in Isabelle, among others:

Using these properties and some others already present in the library, we prove that it is possible to factor a polynomial by factoring its reciprocal and then taking reciprocal of its irreducible factors. To avoid unnecessary computations, we define a function  of type  to do this step for a polynomial which does not have zero as constant part and then assemble everything in a function  of the same type to get a full factorization of any integer polynomial as follows. It satisfies the soundness Theorem 1 from the introduction.
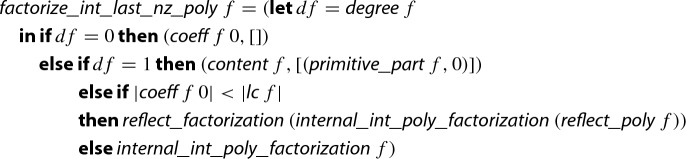

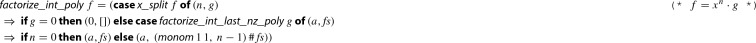


By using Gauss’ lemma we also assembled a factorization algorithm for rational polynomials which just converts the input polynomial into an integer polynomial by a scalar multiplication and then invokes . The algorithm has exactly the same soundness statement as Theorem 1 except that the type changes from integer polynomials to rational polynomials.

Finally, it is worth noting that several of the presented algorithms require polynomial multiplications. However, there is no fast polynomial multiplication algorithm implemented in Isabelle. Indeed, just the naive one is present in the standard library, which is ${{{\mathcal {O}}}}(n^2)$. Thus, we decided to formalize Karatsuba’s multiplication algorithm, which is an algorithm of complexity $\mathcal{O}(n^{\log _2 3})$, to improve the performance of our verified version of the Berlekamp–Zassenhaus algorithm. Karatsuba’s algorithm performs multiplication operation by replacing some multiplications with subtraction and addition operations, which are less costly [16]. We provide a verified implementation for type-based polynomials, e.g., integer polynomials, but we also implement a record-based one for polynomials over $\mathrm {GF}(p)$, cf. Sect. 3. The type-based formalization is valid for arbitrary polynomials over a commutative ring, so we fully replace Isabelle’s polynomial multiplication algorithm by it.

We also tune the GCD algorithm for integer polynomials, so that it first tests whether *f* and *g* are coprime modulo a few primes. If so, we are immediately done, otherwise the GCD of the polynomials is computed. Our experiments shows that this preprocessing is faster than a direct computation of the GCD. Since this heuristic involves a few small primes, all operations in the heuristic are carried out using 64-bit integers.

## Experimental Evaluation

We evaluate the performance of our algorithm in comparison to a modern factorization algorithm—here we choose the factorization algorithm of Mathematica 11.2 [34]. To evaluate the runtime of our algorithm, we use Isabelle’s code generation mechanism [12] to extract Haskell code for . The code generator is designed for partial correctness, i.e., if an execution of the generated code terminates, then the answer will be correct, but termination itself is not guaranteed. Another restriction is that we rely upon soundness of Haskell’s arithmetic operations on integers, since we map Isabelle’s integer types (  ,  , and  ) to Haskell’s integer types (Data.Word.Word32, Data.Word.Word64, and Integer). The resulting code was compiled with GHC version 8.2.1 using the O2 switch to turn on most optimizations. All experiments have been conducted under macOS Mojave 10.14.1 on an 8-core Intel Xeon W running at 3.2 GHz.

**Fig. 1:**
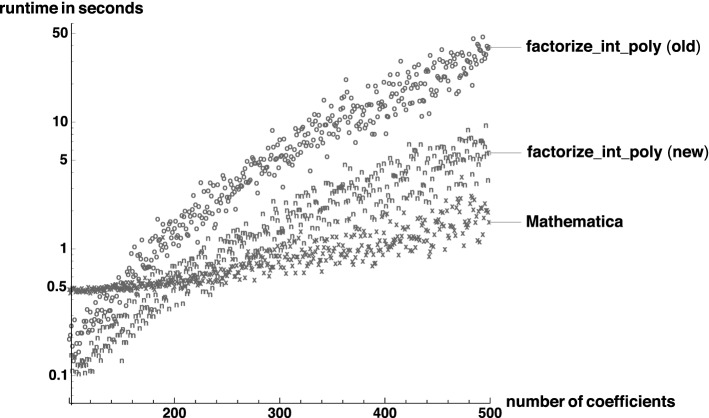
Runtimes compared with Mathematica and the version with no improvements

Figure 1 shows the runtimes of our implementation compared to that of Mathematica on a logarithmic scale. We also include a comparison between the version presented in our previous work [10] and the new one which includes the optimizations explained through this paper. The runtimes are given in seconds (including the 0.5 s startup time of Mathematica), and the horizontal axis shows the number of coefficients of the polynomial. The test suite consists of 400 polynomials with degrees between 100 and 499 and coefficients are chosen at random between $-100$ and 100.

**Table 2:** Impact of individual optimizations

Algorithm	Total runtime (%)
New	100.0
New without GCD heuristic	$+$ 1.2
New without reciprocal polynomials	$+$ 3.3
New without dynamic selection of $\mathrm {GF}(p)$ implementation	$+$ 15.5
New without balanced multifactor Hensel lifting	$+$ 16.7
New without Karatsuba’s multiplication algorithm	$+$ 26.7

As these polynomials have been randomly generated, they are typically irreducible. In this case using a fast external factorization algorithm as a preprocessing step will not improve the performance, as then the preprocessing does not modify the polynomial. We conjecture that the situation could be alleviated by further incorporating an efficient irreducibility test.

Besides making a global comparison between the old and the new algorithm, we also evaluate several different optimizations separately. The results are presented in Table 2, where a row “new without *opt*” indicates a configuration, where only optimization *opt* has been disabled in the new implementation. The time is given relative to the implementation “new” which includes all optimizations and requires around 14 min to factor all 400 example polynomials. The table does not list all optimizations of this paper, since some of them could not easily be disabled in the generated code. In particular, all configurations use the same variant of the binary Hensel lifting algorithm, which considerably differs from the binary Hensel lifting of the old implementation. The results show, that in particular the dynamic selection of the $\mathrm {GF}(p)$ implementation, the balancing of multifactor Hensel lifting, and the improved polynomial multiplication algorithm are significant improvements.

Profiling revealed that for the 400 random example polynomials, most of the time is spent in the Berlekamp factorization, i.e., in step 6 of Algorithm 1, or more precisely in Step 3 of Algorithm 2, the computation of the basis via Gauss–Jordan elimination. Interestingly, the exponential reconstruction algorithm in step 9 does not have any significance on these random polynomials, cf. Table 3.

Nevertheless we remark that this situation can dramatically change on non-random polynomials, e.g., on polynomials constructed via algebraic numbers. For instance when computing the minimal integer polynomial that has $\sum _{i=1}^6 \root 3 \of {i}$ as root, 87.3% of the overall time is spent in the reconstruction algorithm; and for $\sum _{i=1}^7 \root 3 \of {i}$ we had to abort the computation within the reconstruction phase. Note that even Mathematica does not finish the computation of the latter minimal polynomial within a day. As a possible optimization, the exponential reconstruction phase can be replaced by van Hoeij’s fast reconstruction algorithm based on lattice-reduction [14], which is implemented in Maple 2017.3 [25]. Although Maple is only 20 % faster than Mathematica when factoring the 400 random polynomials, it can compute the minimal polynomial within a second, in contrast to the timeout of Mathematica. However, a soundness proof of van Hoeij’s algorithm is much more involved.

**Table 3:** Profiling results

Step	Amount of total runtime (%)
Berlekamp factorization	75.45
Hensel lifting	22.79
Square-free factorization	0.65
Find suitable prime	0.63
Determine factor bound	0.38
Remaining parts	0.09

## Summary

We formalized the Berlekamp–Zassenhaus algorithm for factoring univariate integer polynomials. To this end we switched between different representations of finite fields and quotient rings with the help of locales, the transfer package and local type definitions. The generated code can factor large polynomials within seconds. The whole formalization consists of 21320 lines of Isabelle and took about 17 person months of Isabelle experts. As far as we know, this is the first formalization of an efficient polynomial factorization algorithm in a theorem prover.

Most of the improvements mentioned as potential future work in our previous conference paper [10] have now been formalized and are integrated in the development, but there still remain some possibilities to extend the current formalization for optimizing the factorization algorithm even further. For instance, one can consider using the Cantor–Zassenhaus algorithm [8] for finite-field factorization, although its formalization would be more intricate (indeed, it is a probabilistic algorithm).

## Footnotes

1 Our algorithm starts with step 4, so that section numbers and step-numbers coincide.

2 A formalization of the ring ${\mathbb {Z}}/{p}{\mathbb {Z}}$ is already present in  as a locale  . In principle we could reuse results from the library by proving a connection between the locale and our class; however, as the resulting proofs became slightly longer than direct proofs, we did not use this library.

3 We would like to have introduced this abbreviation also in Isabelle. However, we are not aware of how to do this, since the  keyword does not allow specifying type constraints such as .

4 In this paper we use the conventional notations $f \equiv g \quad (mod m)$ and $f \mathbin {mod }m$. In the formalization these notions are defined as  and  respectively.

5 The preliminary version [10] of this paper does not require such an abstraction layer since there we always implement $\mathrm {GF}(p)$ via integers.

6 Knuth gives a brief outline of a proof, but he also classifies the exercise as a problem of moderate complexity that may involve more than two hours’ work to solve it on paper.

7 Indeed, one can reduce the degree bound to half of the degree of *f* if one uses a slightly more complex reconstruction algorithm which sometimes considers the complement of the selected factors. We did not investigate the trade-off between the two alternatives.

8 One might transfer the type-based Lemma 21 to integer polynomials, in order to use it within the inductive proof of Lemma 22. However, the current proof of Lemma 22 does not rely upon Lemma 21.

9 Perhaps our quadratic version of Hensel lifting is faster than the iterated one-step version since we did not integrate (and prove) optimizations (iii) and (iv) of Miola and Yun [28, Sect. 2.4].

10 Although  does not support pattern matching, we prefer to use pattern matching in the presentation.
